# Association between patient-reported outcomes and exercise test outcomes in patients with COPD before and after pulmonary rehabilitation

**DOI:** 10.1186/s12955-020-01505-x

**Published:** 2020-09-05

**Authors:** Roy Meys, Anouk A. F. Stoffels, Sarah Houben-Wilke, Daisy J. A. Janssen, Chris Burtin, Hieronymus W. H. van Hees, Frits M. E. Franssen, Bram van den Borst, Emiel F. M. Wouters, Martijn A. Spruit, on behalf of the BASES-consortium

**Affiliations:** 1 Department of Research and Development, CIRO, Hornerheide 1, 6085NM Horn, the Netherlands; 2 Department of Respiratory Medicine, Maastricht University Medical Centre, NUTRIM School of Nutrition and Translational Research in Metabolism, Maastricht, The Netherlands; 3 Department of Pulmonary Diseases, Radboud UMC Dekkerswald, Nijmegen, the Netherlands; 4 Department of Health Services Research, Care and Public Health Research Institute, Faculty of Health Medicine and Life Sciences, Maastricht University, Maastricht, the Netherlands; 5 Reval Rehabilitation Research, Biomedical Research Institute, Faculty of Rehabilitation Sciences, Hasselt University, Diepenbeek, Belgium

**Keywords:** COPD, Patient-reported outcome measures, Exercise test, Pulmonary rehabilitation, Quality of life

## Abstract

**Background:**

Over the years, the scope of outcomes assessment in chronic obstructive pulmonary disease (COPD) has broadened, allowing for the evaluation of various patient-reported outcomes (PROs). As it still remains unclear whether and to what extent PROs mirror the exercise performance of patients with COPD, the current study aimed to assess the association between different exercise test outcomes and PROs, before and after pulmonary rehabilitation (PR).

**Methods:**

Correlations between PROs used to describe health-related quality of life (HRQoL), mood status, level of care dependency and dyspnea in patients with COPD and commonly used laboratory- and field-based exercise test outcomes were evaluated in 518 individuals with COPD attending PR.

**Results:**

Overall, correlations between PROs and exercise test outcomes at baseline were statistically significant. The correlation between modified Medical Research Council (mMRC) dyspnea score and 6-min walking distance (6MWD) was strongest (ρ:-0.65; *p*<0.001). HRQoL related PROs showed weak correlations with exercise outcomes at baseline. Moderate correlations were found between St George’s Respiratory Questionnaire total score and 6MWD (*r*:-0.53; *p*<0.001) and maximal workload achieved during cardiopulmonary exercise testing (*ρ*:-0.48; *p*<0.001); and between Clinical COPD Questionnaire (CCQ) total score and 6MWD (*r*:-0.48; *p*<0.001) and maximal workload (*ρ*:-0.43; *p*<0.001). When significant, correlations between changes in exercise test outcomes and changes in PROs after PR were generally very weak or weak. The highest correlation was found between changes in CCQ total score and changes in 6MWD (ρ: − 0.36; *p*<0.001).

**Conclusions:**

PROs and exercise test outcomes, although significantly correlated with each other, assess different disease features in patients with COPD. Individual PROs need to be supported by additional functional measurements whenever possible, in order to get a more detailed insight in the effectiveness of a PR program.

**Trial registration:**

Netherlands Trial Register (NL3263/NTR3416). Registered 2 May 2012.

## Background

Patients with chronic obstructive pulmonary disease (COPD), a highly-prevalent chronic lung disease, frequently suffer from symptoms of dyspnea, exercise intolerance, an impaired mood status and a reduced health status [1–3]. These features are typically weakly related to the degree of lung function impairment [4]. Therefore, the use of additional assessments such as exercise tests and patient-reported outcomes (PROs) has been advocated [3, 5, 6]. Appraisal of these extra-pulmonary features is necessary to better understand the patients’ daily needs or problems, to identify possible treatable traits for integrated COPD care programs, and to evaluate its efficacy [7].

Several laboratory- and field-based exercise tests can be performed to measure exercise performance, which is typically affected in patients with COPD [3, 8], due to a downward spiral of dyspnea, disability and physical inactivity [9]. Important aspects from the patient’s perspective like health-related quality of life (HRQoL), dyspnea, anxiety, depression, and the level of care dependency, all of which have a direct impact on daily life [10], are measured using PROs.

Punekar and colleagues systematically reviewed the strength of the available evidence supporting correlations between the outcomes of different exercise tests and PROs most commonly used to assess HRQoL and dyspnea [11]. They concluded that only a limited amount of studies have focused on the correlations between exercise test outcomes and PROs in patients with COPD. The available evidence indicates a very weak to moderate negative correlation between 6-min walking distance (6MWD) and HRQoL, measured with the St. George’s Respiratory Questionnaire (SGRQ). The relationship between PROs for dyspnea and 6MWD showed contrasting results, with both moderate to strong positive and negative correlations being reported [11]. So, it still remains unclear whether and to what extent PROs mirror the exercise performance of patients with COPD. It seems reasonable to hypothesize that other exercise test outcomes than 6MWD may be stronger correlated with different PROs. For example, disease-specific questionnaires like the Clinical COPD Questionnaire (CCQ) and the COPD Assessment Test (CAT) focus more on functional impairments and symptoms related to COPD and may therefore be more closely associated with exercise test outcomes in patients with COPD.

Pulmonary rehabilitation (PR) reduces dyspnea, increases exercise capacity, and improves HRQoL in individuals with COPD [6]. Exercise training is a major component of PR and therefore exercise test outcomes are consistently used to assess the individual patient’s response to PR [12–17]. Nevertheless, improvements in exercise performance after PR do not necessarily lead to a concurrent decrease in symptoms in patients with COPD and vice versa [18]. Therefore, the question remains whether changes in exercise test outcomes after PR translate into changes in disease-specific PROs.

In this observational study, we aimed to assess the association between different exercise test outcomes and PROs most commonly used to describe HRQoL, anxiety, depression and disease-specific symptoms, such as dyspnea, in patients with COPD before and after PR. A priori, we hypothesized that the correlation between PROs for dyspnea and HRQoL and exercise test outcomes would be statistically significant, but that there would be no strong or very strong association. Furthermore, it was expected that improvements in exercise test outcomes after PR showed weak correlations with changes in PROs in patients with COPD.

## Methods

### Study design and participants

The current study is a retrospective analysis of the *‘COPD, Health status and Comorbidities’* (Chance) study, Netherlands Trial Register NTR3416 [19]. The Medical Ethical Committee of the Maastricht University Medical Centre+ (MEC 11–3-070) approved this trial, which conformed to the ‘Declaration of Helsinki’ as amended most recently by the 64th WMA General Assembly, Fortaleza, Brazil, October 2013 [20]. The Medical Research Involving Human Subjects Act (WMO) does not apply for the secondary analysis of the Chance study. Therefore, an additional official approval of this secondary analysis by the Medical Ethical Committee is not required (MEC letter 2019–0987).

Patients with mild to very severe COPD were recruited before the start of a comprehensive PR program at CIRO in Horn, The Netherlands [21]. Patients between the age of 40 and 85 years with a diagnosis of COPD according to GOLD guidelines [22] were eligible. The protocol and part of the results of the Chance-study have been published before [1, 4, 10, 15, 19, 23–26]. All patients gave written informed consent prior to inclusion in the study.

### PR program

PR took place inpatient (8 weeks, 5 sessions per week; total of 40 sessions) or outpatient (8 weeks, 3 sessions per week, followed by 8 weeks, 2 sessions per week; total of 40 sessions), in line with the 2013 American Thoracic Society & European Respiratory Society Statement [4]. Extensive pre- and post-PR assessments were performed, as described before [19].

### Measurements

Demographics, body mass index (BMI), body composition (fat-free mass index) [27], smoking history were assessed, as part of standard care. Lung function was determined with standardized spirometry equipment of Masterlab (CareFusion, Hoechberg, Germany) [28].

To evaluate HRQoL, three disease-specific PROs, the CAT (range 0–40 points) [29], the CCQ (range 0–6 points) [30] and the COPD-specific version of the SGRQ (range 0–100 points) [31] were assessed in all participants. Mood status was measured with the Hospital Anxiety and Depression scale (HADS; range 0–21 points) [32]. Higher scores are equivalent to a decreased HRQoL and/or increase in symptoms of anxiety or depression, respectively. The mMRC dyspnea scale was used to establish functional impairment due to dyspnea [33]. The level of care dependency was determined at baseline with the Care Dependency Scale (CDS; range 15–75 points) with a lower score representing a higher level of care dependency [34].

The 6-min walking test (6MWT) [35], cardiopulmonary exercise test (CPET; only at baseline) [36] were used to assess exercise capacity. Exercise tolerance was determined as cycle endurance time (CET) during the constant work rate cycle test (CWRT) [37]. Functional mobility was measured with the Timed ‘Up and Go’ (TUG) test [15, 17]. Isokinetic quadriceps muscle function (i.e. strength and endurance/total work) was determined using a Biodex System 4 Pro (Biodex Medical Systems Inc., New York, USA) [38].

### Statistical analyses

Analyses were performed using SPSS software (statistical package for the social sciences) for Windows (version 25.0). Results are presented as mean and standard deviation (SD), median and interquartile range (IQR), and/or proportions, as appropriate. Continuous variables were tested for normality. Differences at baseline between completers and non-completers were analyzed using independent samples T-tests or Mann-Whitney U tests. Correlations between PROs and exercise test outcomes were analyzed using Scatter plots and Pearson’s or Spearman’s correlations, as appropriate. The strength of correlations has been classified according to British Medical Journal guidelines, which regard significant correlation coefficients of 0–0.19 as very weak, 0.2–0.39 as weak, 0.4–0.59 as moderate, 0.6–0.79 as strong, and 0.8–1 as very strong [39]. A priori, the level of significance was set at ≤0.01.

## Results

A total of 518 patients (55.6% male, age 64.1 ± 9.1 years) volunteered to participate and attended the pre-PR assessment. The mean baseline 6MWD was 424 ± 124 m and 25.1% of the patients had a 6MWD below 350 m [40] and in 74.7% of the patients, quadriceps muscle strength was less than 80% of the predicted value [41]. The PROs showed a high degree of dyspnea (80.7% with mMRC dyspnea grade of two or higher) [22], anxiety (34.8% with ≥10 points) [32], depression (33.4% with ≥10 points) [32], care dependency (28.5% with CDS total score of ≤68 points) [25], and an impaired HRQoL (81.9% with a SGRQ total score of ≥44 points; 75.0% with a CAT total score of ≥18 points; 76.7% CCQ total score of ≥1.9 points) [22]. Baseline characteristics, exercise test outcomes and PROs at baseline are presented in Table 1.

**Table 1:** Patient characteristics, patient-reported outcomes and exercise test outcomes at baseline

	Whole group	*n*	Completers	*n*	Non-completers	*n*
	*N* = 518		*N* = 419		*N* = 99	
Patient characteristics						
Gender, male (%)	288 (55.6)	518	232 (55.4)	419	56 (56.6)	99
Age, years	64.1 ± 9.1	518	64.3 ± 8.8	419	63.2 ± 10.3	99
Current smoker, n (%)	114 (22.1)	518	79 (18.9)	419	35 (35.4)^*^	98
Pack years, n	40.0 (30.0–50.0)	518	40.0 (30.0–50.0)	403	40.0 (30.0–51.0)	93
BMI, kg/m^2^	26.2 ± 5.8	518	26.2 ± 5.7	419	26.2 ± 6.3	99
FFMI, kg/m^2^	17.0 ± 2.5	499	17.0 ± 2.4	405	17.0 ± 2.6	94
FEV_1_, L	1.29 ± 0.60	518	1.30 ± 0.60	419	1.26 ± 0.60	99
FEV_1_% predicted	48.6 ± 20.0	518	48.9 ± 20.0	419	47.3 ± 20.1	99
FEV_1_ / FVC, %	37.5 ± 12.2	518	37.3 ± 12.1	419	38.4 ± 12.9	99
mMRC-score (0/1/2/3/4), %	2/17/38/25/18	512	2/17/40/22/18	414	0/15/27/36/22	98
GOLD classification (I/II/III/IV), %	7/36/37/20	518	8/36/35/21	419	6/33/43/17	99
GOLD classification (A/B/C/D), %	3/20/5/72	518	2/22/5/71	419	5/12/5/78	99
Oxygen saturation, %	94.6 (92.7–96.0)	510	94.6 (92.8–96.0)	414	94.0 (92.0–96.0)	96
LTOT, n (%)	125 (24.1)	518	104 (24.8)	419	21 (21.2)	96
Patient-reported outcomes						
mMRC score, points	2.4 ± 1.0	512	2.4 ± 1.0	414	2.7 ± 1.0	98
SGRQ-C total score, points	61.1 ± 17.4	504	60.1 ± 17.1	409	65.4 ± 18.1^*^	95
CAT total score, points	21.5 ± 6.6	505	21.5 ± 6.6	410	21.7 ± 6.9	95
CCQ total score, points	2.6 ± 1.0	502	2.6 ± 1.0	409	2.8 ± 1.1	93
HADS-A score, points	7.8 ± 4.5	500	7.5 ± 4.4	407	9.0 ± 4.9^*^	93
HADS-D score, points	7.5 ± 4.3	500	7.4 ± 4.2	407	8.0 ± 4.9	93
CDS total score, points	72.0 (68.0–75.0)	480	69.7 ± 7.2	389	68.4 ± 7.9	91
Exercise test outcomes						
6MWD, meters	424 ± 124	513	431 ± 124	417	393 ± 123^*^	96
CPET W_max_, W	70.1 ± 34.2	493	70.9 ± 33.7	407	66.6 ± 36.7	86
CPET VO_2peak_, ml/min	1090 ± 414	390	1094 ± 407	316	1071 ± 446	74
CWRT endurance time, seconds	224 (169–327)	477	235 (174–338)	392	199 (149–294)^*^	85
TUG test time, seconds	9.8 (8.5–11.8)	500	9.6 (8.3–11.6)	408	10.2 (8.7–12.7)	92
Quadriceps peak torque, Nm	94.1 ± 36.4	466	94.4 ± 35.9	383	93.5 ± 39.1	83
Quadriceps total work, J	1627 ± 741	465	1641 ± 724	382	1559 ± 815	83

Summary variables are presented as n (%) for discrete variables, mean ± standard deviation for quantitative variables or median (Interquartile range) for skewed variables, * *p*<0.01. ‘n’ represents the total number of sample values per analysis

*Abbreviations*: *BMI* body mass index, *FFMI* Fat Free Mass Index, *FEV1* forced expiratory volume in the first second, *FVC* forced vital capacity, *mMRC* modified Medical Research Council scale, *GOLD* Global Initiative for Chronic Obstructive Lung Disease, *LTOT* Long Term Oxygen Therapy, *mMRC* modified Medical Research Council scale, *SGRQ-C* COPD-specific St. George Respiratory Questionnaire score, *CAT* COPD Assessment Test, *CCQ* Clinical COPD Questionnaire, *HADS-A* Hospital Anxiety and Depression Scale, Anxiety subscale, *HADS-D* Hospital Anxiety and Depression Scale, Depression subscale, *CDS* Care Dependency Scale, *6MWD* 6-min walking distance, *CPET* Cardiopulmonary Exercise Test, *Wmax* maximal achieved workload, *W* Watts, *VO2peak* peak oxygen uptake, *ml = milliliter* min = minute, *CWRT* Constant Work-Rate Test, *TUG* Timed ‘Up and Go’, *Nm* Newtonmeter, *J* Joules

### Correlations between exercise test outcomes and PROs at baseline

**Fig. 1:**
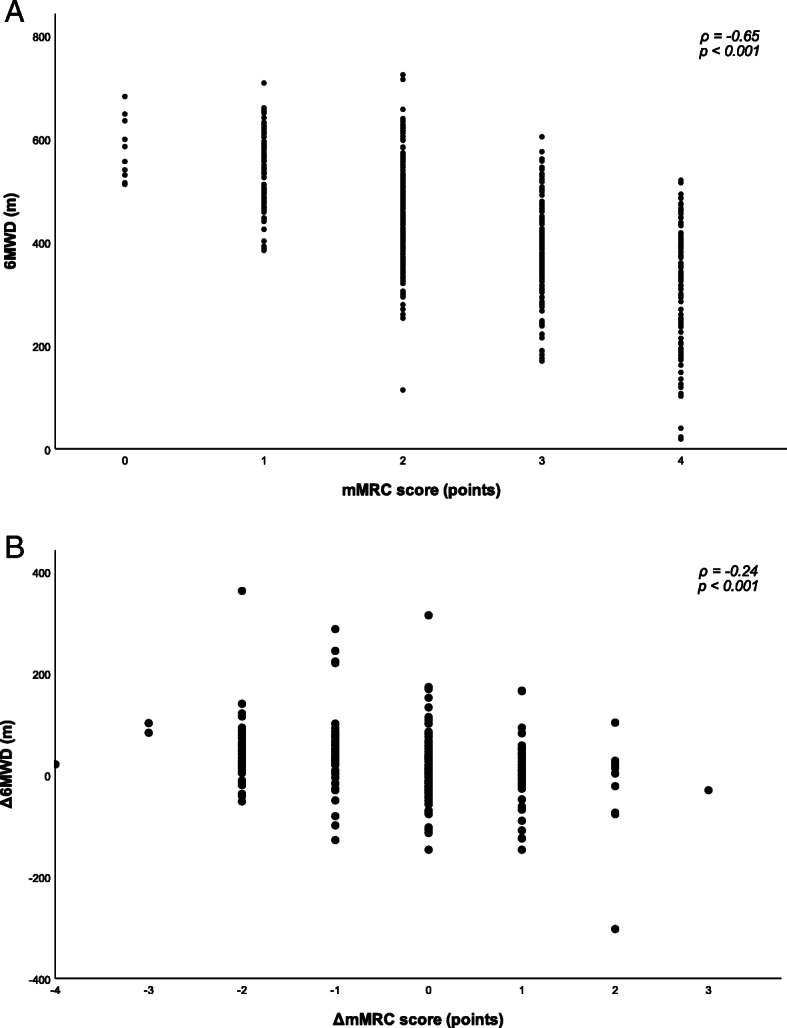
Association between the mMRC score and 6MWD at baseline (**a**) and between changes in mMRC score and changes in 6MWD after PR (**b**)

Overall, correlations between PROs and exercise test outcomes at baseline were statistically significant (Table 2). Of these, the correlation between mMRC score and 6MWD was the strongest (*ρ*: −0.65; *p*<0.001), which is visually presented in Fig. 1. A moderate correlation was found between mMRC score and CPET maximum workload (W_max_; *ρ*: −0.54; *p*<0.001), CPET peak oxygen uptake (VO_2peak_; *ρ*: −0.40; *p*<0.001), TUG time (*ρ*: 0.49; *p*<0.001), quadriceps total work (*ρ*: −0.43; *p*<0.001), respectively.

**Table 2:** Correlations between exercise test outcomes and PROs at baseline

	6MWD (m)	CPET (W_max_)	CPET (VO_2peak_)	CWRT (t)	TUG (t)	Q. Peak torque	Q. Total work
mMRC score	−0.65^*^	−0.54^*^	−0.40^*^	−0.39^*^	0.49^*^	−0.32^*^	−0.43^*^
SGRQ-C total score	−0.53^*^	−0.48^*^	−0.31^*^	−0.35^*^	0.41^*^	−0.26^*^	−0.38^*^
CAT total score	−0.37^*^	−0.30^*^	−0.21^*^	−0.21^*^	0.27^*^	−0.23^*^	−0.26^*^
CCQ total score	−0.48^*^	−0.43^*^	−0.30^*^	−0.29^*^	0.34^*^	−0.25^*^	−0.34^*^
HADS-A score	−0.25^*^	−0.20^*^	−0.10	−0.09	0.21^*^	−0.16^*^	−0.22^*^
HADS-D score	−0.27^*^	−0.22^*^	−0.06	−0.08	0.26^*^	−0.11	−0.20^*^
CDS total score	0.50^*^	0.40^*^	0.25^*^	0.24^*^	−0.43^*^	0.28^*^	0.34^*^

Correlations are reported as Pearson’s *r* or, in the case of ordinal and/or skewed variables or variables with significant outliers, as Spearman’s *ρ*; * *p*<0.001

*Abbreviations*: *mMRC*, modified Medical Research Council scale; *SGRQ-C*, COPD-specific St. George Respiratory Questionnaire; *CAT*, COPD Assessment Test; *CCQ*, Clinical COPD Questionnaire; *HADS-A*, Hospital Anxiety and Depression Scale, Anxiety subscale; *HADS-D*, Hospital Anxiety and Depression Scale, Depression subscale; *CDS*, Care Dependency Scale; *6MWD*, 6-min walking distance; *CPET*, Cardiopulmonary Exercise Test; *Wmax*, maximal achieved workload; *VO2peak*, peak oxygen uptake; *t*, time; *CWRT*, Constant Work-Rate Test; *TUG*, Timed ‘Up and Go’ test; *Q*, Quadriceps muscle

**Fig. 2:**
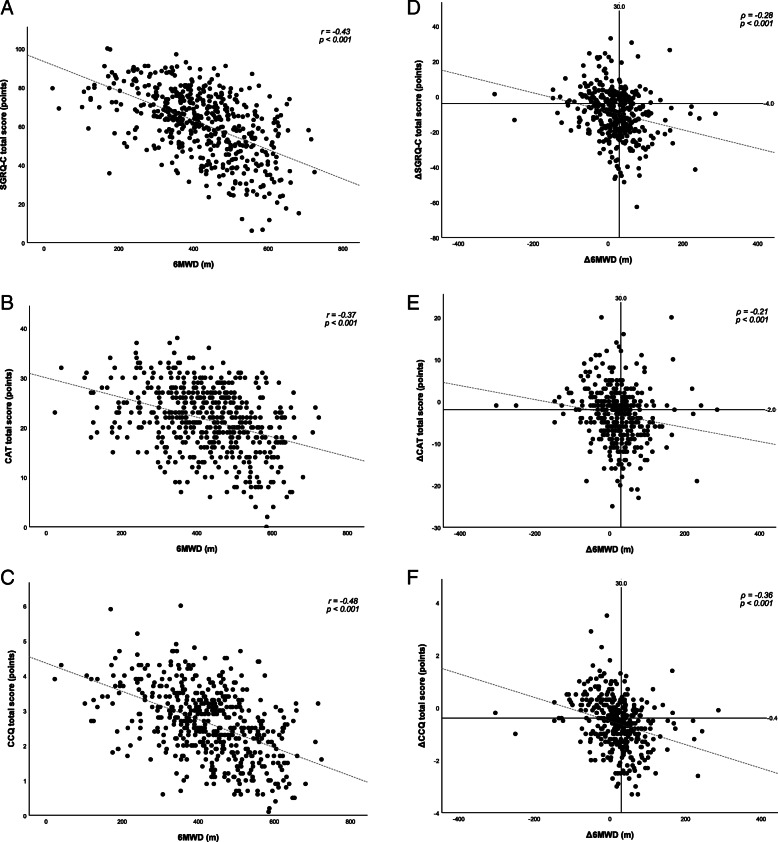
Left: Association between the SGRQ-C score (**a**), CAT score (**b**), CCQ score (**c**), and the 6MWD. Right: association between the change in SGRQ-C score (**d**), CAT score (**e**), CCQ score (**f**), and the change in 6MWD after PR. On the X-axis the MCID of 30 m for the 6MWT [42] is marked, on the Y-axis the MCIDs for SGRQ-C (−4.0), CAT (−2.0) and CCQ score (−0.4) are marked [10, 43]. MCID, minimal clinically important difference

HRQoL PROs showed weak correlations with exercise outcomes at baseline. Moderate correlations were only found between SGRQ-C and 6MWD (*r*: −0.53; *p*<0.001) and CPET maximum workload (*ρ*: −0.48; *p*<0.001) and between CCQ and 6MWD (*r*: −0.48; *p*<0.001) and CPET maximum workload (*ρ*: −0.43; *p*<0.001). See Fig. 2 for a scatter plot illustrating the relationship between HRQoL PROs and 6MWD. CDS score was significantly correlated with all exercise test outcomes, with correlations ranging from 0.24 (CWRT cycle endurance time) to 0.50 (6MWD). Both HADS-D and HADS-A showed non-significant or very weak to weak correlations with all exercise test outcomes.

### Correlations between changes in exercise test outcomes and changes in PROs after PR

Four hundred nineteen patients completed the PR program. Completers and non-completers were comparable with respect to baseline characteristics (Table 1). Only the amount of current smokers was significantly higher in the non-completer group (*p*<0.001). All PROs and exercise test outcomes changed significantly after PR (Table S1). When significant, correlations between changes in exercise test outcomes and changes in PROs were generally very weak or weak. The highest correlation, being classified as weak, was found between ΔCCQ and Δ6MWD (ρ: −0.36; *p*<0.001; Fig. 2). Changes in other HRQoL PROs demonstrated similar association with changes in exercise test outcomes (Table 3). Changes in quadriceps peak muscle strength were not correlated with changes in any of the PROs.

**Table 3:** Correlations between changes in exercise test outcomes and changes in PROs (pre vs. post PR)

		Δ6MWD (m)	ΔCWRT (t)	ΔTUG (t)	Δ Q. Peak torque	Δ Q. Total work
ΔmMRC score	*ρ*	−0.24^*^	−0.08	0.19^#^	−0.08	−0.15
ΔSGRQ-C total score	*ρ*	−0.28^*^	−0.29^*^	0.11	−0.03	−0.10
ΔCAT total score	*ρ*	−0.21^*^	−0.24^*^	0.06	0.03	−0.08
ΔCCQ total score	*ρ*	−0.36^*^	−0.33^*^	0.15^#^	−0.03	−0.16^#^
ΔHADS-A score	*ρ*	−0.19^*^	−0.17^#^	0.12	−0.04	−0.07
ΔHADS-D score	*ρ*	−0.15^#^	−0.21^*^	0.16^#^	0.01	−0.08

Spearman’s *ρ* is reported since all exercise outcomes changes showed significant outliers; * *p*<0.001; ^#^ *p* < 0.01

*Abbreviations*: *mMRC*, modified Medical Research Council scale; *SGRQ-C*, COPD-specific St. George Respiratory Questionnaire score; *CAT*, COPD Assessment Test; *CCQ*, Clinical COPD Questionnaire; *HADS-A*, Hospital Anxiety and Depression Scale, Anxiety subscale; *HADS-D*, Hospital Anxiety and Depression Scale, Depression subscale; *6MWD*, 6-min walking distance; *t*, time; *CWRT*, Constant Work-Rate Test; *TUG*, Timed ‘Up and Go’ test; *Q* Quadriceps muscle

## Discussion

This study demonstrates that PROs and exercise test outcomes are associated to some extent in patients with mild to very severe COPD, but, in general, these correlations are weak to moderate. A strong relationship was merely found between the severity of dyspnea (mMRC) and distance covered in the 6MWT at baseline. In the current study, dyspnea tended to indicate at least moderate negative correlations with exercise test outcomes at baseline, suggesting that exercise performance decreases as dyspnea scores increase. However, these associations attenuated considerably or even became non-significant once the changes in dyspnea were correlated with changes in exercise test outcomes following PR, indicating that an improvement in exercise performance after PR does not necessarily imply that self-reported breathlessness decreases concurrently, like shown before [18]. As a side remark, it is important to note that correlations between changes in parameters are always lower than cross-sectional correlations. After all, the measurement error is included twice (pre vs. post) in the analysis, which always results in a weaker signal [44].

While the mMRC-scale is a unidimensional method to quantify only dyspnea, there are several multidimensional disease-specific PROs, which assess not only dyspnea but also other symptoms and perceived HRQoL in COPD [1]. Of these HRQoL PROs (CAT, CCQ, SGRQ), their association with exercise test outcomes was weak to moderate, indicating that no single exercise test accurately reflects HRQoL (or the other way around), proving that HRQoL is indeed a multi-dimensional concept that includes domains related to physical, mental, emotional, and social functioning. Overall, these results support the findings by Punekar et al. [11] who showed that generally there was a very weak to moderate negative correlation between the 6MWD and the SGRQ.

While guidelines on the diagnosis and treatment of COPD have intensively stated that the assessment of disease severity is substantially improved by using functional criteria [22], such as exercise capacity, the current study demonstrates that the variance in PROs can only be partially explained by attributes related to exercise performance. So, despite the fact that PROs for HRQoL, dyspnea, anxiety, depression and the level of care dependency are crucial when evaluating the disease severity and effectiveness of a treatment in COPD, it is justified to conclude that these PROs assess features not measured by exercise tests. Consequently, if we solely use a few outcome measures (for example, walking distance or HRQoL) to evaluate performance after PR, the clinical complexity and multidimensional aspect of PR in patients with COPD appears to be ignored [18].

In our study, the 6MWD showed the strongest relationship with important clinical PROs, underlining the fact that the 6MWT indeed seems to play a key role in evaluating *functional* exercise capacity [14]. Since the 6MWT is self-paced, test outcomes are likely to be affected by a patient’s mental and emotional status [3].

### Limitations

Patients were solely recruited in a specialized PR centre, resulting in a selected group of COPD patients. This should be considered when applying results to other COPD samples. Furthermore, by quantifying the associated exercise limitation, a mMRC-score of 4 reflects the most disabled COPD patients who are not always able to perform a symptom-limited CPET, as a result of their dyspnea. In the current study, patients unable to perform a CPET and, concurrently, a CWRT were automatically excluded from the correlation analysis, since they did not present any values for both exercise tests, possibly affecting the correlation coefficients.

## Conclusions

In conclusion, we have found that patient-reported outcomes and exercise test outcomes, although significantly correlated with each other, assess different disease features in patients with COPD. Therefore, it can be stated that relevant features from the patient’s perspective like HRQoL, anxiety, depression, and the level of care dependency are not an accurate reflection of a patient’s exercise capacity. The only exception to this seems to be dyspnea, the only PRO that tended to imply at least moderate association with exercise test outcomes. We would like to highlight the complexity of evaluating the effectiveness of a personalized PR program, in which we note that changes in PROs and changes in exercise test outcomes correlate poorly. Indeed, improvements in exercise capacity obtained after PR do not necessarily result in alterations in PROs in patients with COPD. Individual PROs need to be supported by additional functional measurements whenever possible, in order to get a more detailed insight in the effectiveness of a PR program.

## Supplementary information

**Additional file 1: Table S1.** Changes in PROs and exercise test outcomes after PR.

## Data Availability

The datasets generated during and/or analyzed during the current study are available from the Board of Directors of CIRO on reasonable request and following the CIRO’s data request policy.

## Abbreviations

6MWD: Six-minute Walking Distance
6MWT: Six-minute Walking Test
BMI: Body Mass Index
CAT: COPD Assessment Test
CCQ: Clinical COPD Questionnaire
CDS: Care Dependency Scale
COPD: Chronic Obstructive Pulmonary Disease
CPET: Cardiopulmonary Exercise Test
CWRT: Constant Work Rate (cycle) Test
FEV_1_: Forced Expiratory Volume in One Second
HADS: Hospital Anxiety and Depression Scale
HRQoL: Health-Related Quality of Life
IQR: Interquartile Range
mMRC: modified Medical Research Council
MEC: Medical Ethics Committee
PR: Pulmonary Rehabilitation
PRO: Patient-reported Outcome
SD: Standard Deviation
SGRQ-C: St George’s Respiratory Questionnaire (COPD-specific version)
TUG: Timed Up and Go
VO_2peak_: Peak Oxygen Uptake
W_max_: Maximal Workload
WMO: Medical Research Involving Human Subjects Act
